# Meditation on *OM*: Relevance from ancient texts and contemporary science

**DOI:** 10.4103/0973-6131.66771

**Published:** 2010

**Authors:** Sanjay Kumar, HR Nagendra, NK Manjunath, KV Naveen, Shirley Telles

**Affiliations:** Department of Yoga Research, Indian Council of Medical Research Centre for Advanced Research in Yoga and Neurophysiology, SVYASA, Bangalore, India

**Keywords:** *Om*, *upanisads*, patanjali’s yoga *sutras*, autonomic variables

## Abstract

**Background:**

In Indian scriptures the sacred syllable *Om* is the primordial sound from which all other sounds and creation emerge which signifies the Supreme Power.

**Aims:**

To explore the significance of the syllable *OM* from ancient texts and effects of OM meditation in contemporary science.

**Descriptions from ancient texts:**

The descriptions of Om have been taken from four Upanisads (*Mundaka, Mandukya, Svetasvatara*, and *Katha*), the *Bhagvad Gita*, and Patanjali’s Yoga *Sutras*.

**Scientific studies on *Om*:**

Autonomic and respiratory studies suggest that there is a combination of mental alertness with physiological rest during the practice of *Om* meditation. Evoked potentials studies suggest a decrease in sensory transmission time at the level of the auditory association cortices, along with recruitment of more neurons at mesencephalic-diencephalic levels.

**Conclusions:**

It is considered that a person who realizes *Om*, merges with the Absolute. Scientific studies on *Om* suggest that the mental repetition of *Om* results in physiological alertness, and increased sensitivity to sensory transmission.

## REFERENCES TO *OM* IN THE SCRIPTURES

### General

Symbolism has a place in spirituality. Healing methods based on altered states of consciousness are common in spiritual or shamanic traditions but escape neuroscientific explanations based on classical cognition.[1] They are described here as a “perceptual-cognitive-symbolic” characteristic of ordinary states of consciousness. Another channel source of information processing, called “direct-intuitive-nonlocal,” characteristic of nonordinary states of consciousness is required to be introduced for interpretation. The first one is capable of modeling via symbolism and is more culturally bound due to its psycholinguistic features. The second one lacks symbolism; therefore, the first one has more transcultural similarity, though culture-specific transliteration may occur.

Among many symbols used, *Om* is one of the fundamental symbols used in the yoga tradition.

## REFERENCES IN THE UPANISHADS

*Om* is the name or symbol of God (*Ishwara, Brahman*).[2] *Om* covers the whole threefold experience of man. It is the combination of three letters, namely, A, U, and M.[3] “A” represents the physical plane. “U” represents the mental and astral plane, the world of intelligent spirits, and all heavens. “M” represents the whole deep-sleep state, which is unknown even in our wakeful state.[3] This concept has been well described in various Indian scriptures. In *Mandukya Upanishad*, it has been described that *Om* is the syllable of the past, the present, and the future.[4] From the original sound, *Om*, all things become manifest as its extension embodiments.[4]

The analogy in *Mundaka Upanishad* describes that *Om* is the bow; the soul is the arrow; and Brahman is the target. The target is attained by an unerring man. One should become one with the target just like an arrow. This is to become one with the imperishable by eliminating the ideas of the body, ego, *prana*, hence being the self with nothing less than union with the absolute.[5]

*Svetasvatara Upanishad* describes that *Om* is like the fire which though potentially present in firewood is not seen until two sticks are rubbed against each other. The self is like that fire; it is realized by constant awareness of the sacred syllable *Om*. Let the body be the stick that is rubbed and *Om* be the stick that is rubbed against. Then the real nature is realized which is hidden within, just as fire in a sense hidden in the wood.[6]

## REFERENCES IN PATANJALI’S YOGA SUTRAS

*Patanjali’s Yoga Sutras* (PYS) is one of the classical yoga texts in which the explanation about *Om* is well defined.[7] In PYS, there is a single direct mention about *Pranava (Om)*. That is *Tasya vachakah pranavah* (Ch: I; V: 27). This literally means that *pranava* is virtually *Ishwara* or *Om*, where *Ishwara* is the word denoting God. Even though there is only one mention about *Om* in PYS, the definition of *Ishwara* and the attributes are given in PYS Chapter I, Verses 24-26. In *Sutra* 24, it is said *Klesakarmavipakasayairaparaamrstah purusavisesa Isvarah* (Ch: I; V: 24). This means that God is unique, untouched by affliction, acts, and their consequences. In *Sutra* 25, it is said *Tatra niratisayam sarvajnabijam* (Ch: I; V: 25); this means that in God there is the root for endless omniscience. This description is taken further in *Sutra* 26. The *Sutra* says *Purvesamapi guruh kalenanavachchhedat* (Ch: I; V: 26). This means that since *Ishwara* is not limited by time, He is the original guru or the guru of the earliest guru.

Since PYS has described *pranava* (*Om*) as *Ishwara*, it is interesting to note that *Sutra* 28 describes what *sadhana* requires for *Ishwara* realization. *Sutra* 28 states *Tajjapastadarthabhavanam* (Ch: I; V: 28). This means that mental repetition of *Om* (although *Om* is not specifically mentioned) should be carried out while dwelling on its meaning.[8]

## REFERENCES IN THE BHAGVAD GITA

*Bhagvad Gita* describes Krishna’s instructions to Arjuna just before the great war on the battlefield of Kurukshetra.[9] *Om* is the central element in Krishna’s exposition of spiritual life and practice, speaking from his perspective as the infinite being, enumerating his major manifestations and embodiments. The meaning is that *Om* is nothing less than the supreme consciousness; so there can be nothing greater or a subject more important than *Om*. This is illustrated as “One who is engaged in the practice of concentration, uttering the monosyllable *Om* (the *Brahman* or consciousness) who remembers it always, he attains the supreme goal.[9]

### Summary

The sacred syllable *Om* is the primordial sound from which all other sounds and creation emerge. It underlies all phonetic creations. The utterance of *Om*, consisting of the three letters *A, U*, and *M*, covers the whole process of articulation. It is like the sound of a gong that gradually tapers to a point and merges in silence. One who attains *Om*, merges with the Absolute.

Yoga teachings consider the syllable *Om* to be the force behind all thoughts. Either chanting or thinking about *Om* is anecdotally reported to cause a quiet mental state.

## SCIENTIFIC STUDIES ON *OM*

### General

It is generally recognized that experiencing a yoga practice is of great importance for all yoga techniques. The physiological and psychological effects of practicing meditation on *Om* have been studied. In *Om* meditation, the meditators first concentrate on a picture of *Om* and then mentally chant *mantra Om* effortlessly; this finally leads to a state devoid of effort and focusing, and is characterized by blissful awareness.[10]

## STUDIES ON AUTONOMIC AND RESPIRATORY VARIABLES

The autonomic and respiratory variables were studied in seven experienced *Om* meditators (with the experience ranging from 5 to 20 years). Each subject was studied in two types of sessions–meditation (with a period of mental chanting of *Om*) and control (with a period of nontargeted thinking). The meditators showed a statistically significant reduction in the heart rate during meditation compared to the control period. During both types of sessions, there was a comparable increase in the cutaneous peripheral vascular resistance. This was interpreted as a sign of increased mental alertness even while being physiologically relaxed.[11] Subsequently, a comparison study was done to see the physiological effects which reported that when repetition of *Om* was compared with the repetition of *One* in 12 meditators, there was a difference in the autonomic and respiratory responses. Both types of sessions resulted in a decrease in the heart and breath rates, but the repetition of *Om* alone reduced the skin resistance, suggesting a subtle change in the mental state, related to the significance of the syllable.[12]

Yoga *mantras* and prayers have been found beneficial for many physiological and psychological functions of the body.[13] A study was conducted to test whether rhythmic formulae, namely, recitation of the rosary and yoga mantras can synchronize and reinforce inherent cardiovascular rhythms and modify baroflex sensitivity. There were 23 healthy volunteers. It was observed that during both prayers and mantras, there was an increase in the synchronicity of cardiovascular rhythms when they were recited six times a minute. There was also an increase in baroflex sensitivity. These findings suggested that the recitation of the rosary and certain yoga mantras, at specific frequencies, induce favorable psychological and physiological effects.

In summary it was observed that there is cognitive involvement and a combination of mental alertness with physiological rest during the practice of *Om* meditation. Also, various *mantras* and prayers have been found to be useful in inducing a state of psychological and physiological well-being.

## STUDIES ON EVOKED POTENTIALS IN EXPERIENCED MEDITATORS

In an early study, middle latency auditory evoked potentials (0-100 ms range) were examined in seven proficient subjects before and during the practice of *Om* meditation.[14] Such a study helped in understanding how neural processing at various levels could change differently during a meditation practice in which thoughts are focused on a word or phrase without a conscious effort to do so (i.e., meditation on the syllable *Om*). Similar records were also obtained in seven age-matched “naive” subjects before and during a control period which involved sitting with eyes closed, and with no special instructions for focusing their thoughts. However, there was a small but consistent reduction in the peak latency of the Nb wave (the maximum negativity occurring between 35 and 65 ms). This reduction was observed consistently during three repeat sessions of each subject, while the “naive” subjects did not show this change. These results suggest that during meditation, neural processing at the middle latency auditory evoked potentials level does change and the intersubject variability of middle latency auditory evoked potentials precludes using them as the method of choice for assessing the effects of meditation. Hence middle latency auditory evoked potentials demonstrate small, yet significant changes in the neural activity during meditations.

**Figure 1:**
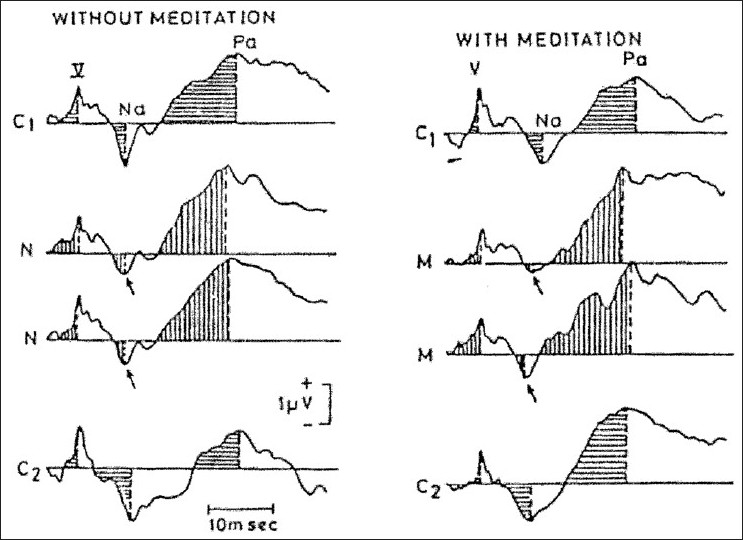
Experienced meditattors versus non-meditators:Importance of motivation. (Typical example of AEP-MLRs during meditation in an experienced subject (left,12 years experience) and a novice (right, 10 days experience). Telles *et al*. 1994, Internal J. Neurosci.)

A subsequent study assessed the effects of OM meditation on middle latency auditory evoked potentials. The experienced meditators showed a significant increase in the peak amplitude of the Na wave (the maximum negative peak between 14 and 18 ms) during the meditation with a significant decrease in the Na wave peak amplitude during the control session [Figure 1]. Hence during mental repetition of a meaningful syllable (*Om*) and of a neutral syllable (*One*), neural changes occurred at the same level (possibly diencephalic) though in opposite directions. To detail this, a significant increase in the peak amplitude of the Na wave occurred during mental chanting of *Om* compared to a significant decrease in the Na wave peak amplitude during a control period of mental chanting of a neutral syllable *One*. It is recognized that an increase in amplitude is correlated with an increase in the number of neurons recruited, whereas a decrease in amplitude has the reverse connotations.

Studies conducted by Telles and Desiraju and Telles *et al*. reported changes in peak latencies of middle latency auditory evoked potential components which reflect changes at subcortical and primary auditory cortex levels. These were suggestive of a decrease in sensory transmission time at these neural levels during the practice of meditation on *Om*.[14,15]

### Summary

Scientific studies on *Om* suggest that the mental repetition of *Om* results in a physiological state at one time characterized by reduced physiological alertness, increased sensitivity as well as synchronicity, as well as changes at specific levels along the auditory pathway suggestive of increased sensitivity to sensory transmission.

## CONCLUSION

The utterance of *Om* consisting of the three letters *A, U*, and *M* covers the whole process of articulation. This sound represents the primal vibration from which all other sounds and creation emerge. It is considered that one who attains *Om*, merges with the Absolute. Scientific studies on *Om* suggest that the mental repetition of *Om* results in physiological alertness, increased sensitivity as well as synchronicity of certain biorhythms, and an increased sensitivity to sensory transmission.
